# Improving Localization in Wireless Sensor Networks for the Internet of Things Using Data Replication-Based Deep Neural Networks

**DOI:** 10.3390/s24196314

**Published:** 2024-09-29

**Authors:** Jehan Esheh, Sofiene Affes

**Affiliations:** EMT Centre (Energy, Materials and Telecommunications), INRS (Institut National de la Recherche Scientifique), Université du Québec, Montréal, QC H5A 1K6, Canada

**Keywords:** data replication, deep neural networks, wireless sensor networks, range-free localization, data augmentation

## Abstract

Localization is one of the most challenging problems in wireless sensor networks (WSNs), primarily driven by the need to develop an accurate and cost-effective localization system for Internet of Things (IoT) applications. While machine learning (ML) algorithms have been widely applied in various WSN-based tasks, their effectiveness is often compromised by limited training data, leading to issues such as overfitting and reduced accuracy, especially when the number of sensor nodes is low. A key strategy to mitigate overfitting involves increasing both the quantity and diversity of the training data. To address the limitations posed by small datasets, this paper proposes an intelligent data augmentation strategy (DAS)-based deep neural network (DNN) that enhances the localization accuracy of WSNs. The proposed DAS replicates the estimated positions of unknown nodes generated by the Dv-hop algorithm and introduces Gaussian noise to these replicated positions, creating multiple modified datasets. By combining the modified datasets with the original training data, we significantly increase the dataset size, which leads to a substantial reduction in normalized root mean square error (NRMSE). The experimental results demonstrate that this data augmentation technique significantly improves the performance of DNNs compared to the traditional Dv-hop algorithm at a low number of nodes while maintaining an efficient computational cost for data augmentation. Therefore, the proposed method provides a scalable and effective solution for enhancing the localization accuracy of WSNs.

## 1. Introduction

A wireless sensor network (WSN) is a type of network consisting of sensor nodes that communicate wirelessly with each other [1]. These sensor nodes are designed to be low-cost, energy-efficient, and capable of reconfiguring themselves as needed. WSNs are self-organizing, meaning the nodes autonomously form a network within a designated area using wireless communication. However, these nodes have limitations in terms of computing power, storage capacity, and data transmission. Despite these constraints, WSN technology offers numerous advantages, including cost-effectiveness, scalability, reliability, and flexibility [2], making it suitable for a wide range of applications, such as environmental monitoring, healthcare, and industrial automation [3,4,5,6,7].

In typical WSN deployments, sensor nodes are often placed randomly. While GPS technology provides the most accurate and reliable method for determining the exact positions of these nodes, equipping each sensor node with a GPS module presents several challenges.

Firstly, the cost of integrating GPS into every sensor node becomes prohibitively expensive, especially for large-scale networks. Secondly, GPS modules have high energy consumption, which is problematic for sensor nodes that are designed to operate with minimal power. Due to these limitations, using GPS for all sensor nodes in a WSN is often impractical.

One solution to the GPS issue in wireless sensor networks (WSNs) is to use a few sensor nodes equipped with localization modules to help determine the positions of other nodes. This technique is known as node localization. In this approach, the sensor nodes with localization modules are called beacon nodes. These beacon nodes know their exact locations, while the other nodes, which do not know their locations, are referred to as unknown nodes. Using the location information from the beacon nodes, the network can estimate the positions of the unknown nodes. This method reduces both cost and power consumption as only a small number of nodes need to be equipped with the more expensive, high-power localization modules.

Node localization can be categorized into range-based and range-free techniques. Range-based algorithms have been developed using various localization methods, each differing in terms of accuracy and cost. Several measurement techniques can be used for position estimation, such as time difference of arrival (TDOA) [8], angle of arrival (AOA) [9], received signal strength (RSS) [10], and time of arrival (TOA) [11]. While these methods offer high accuracy, they often require additional expensive hardware, which raises the overall cost of the system.

In contrast, range-free localization algorithms, such as Centroid [12], Distance Vector-Hop (Dv-hop) [13], and Amorphous [14], offer simpler and more cost-effective alternatives. In the Centroid method, each unknown node estimates its position by averaging the coordinates of nearby anchor nodes. This article focuses on the Dv-hop method, a typical range-free localization technique. The Dv-hop algorithm operates in three phases: first, it identifies the connectivity between nodes, then estimates the number of hops to each anchor node, and finally calculates the unknown node’s position. Amorphous [14], an extension of the Dv-hop algorithm, introduces more sophisticated techniques for estimating distances and locating unknown nodes. Although these range-free methods do not require additional hardware, they are generally less accurate than range-based approaches.

Recently, researchers have begun exploring machine learning techniques to determine the positions of unknown nodes in WSNs. These methods use available data, such as distances or positions, to predict node locations. Machine learning models, including support vector machines (SVMs) [15], neural networks (NNs) [16], and neural network ensembles (NNEs) [17], have been applied to this task. However, these models often face challenges such as overfitting, especially when trained on small datasets. To improve localization accuracy and prevent overfitting, expanding the training dataset with more diverse and higher-quality data is essential.

The contributions of this article are presented as follows:

- A deep neural network (DNN) is implemented to improve the accuracy of unknown node locations in WSNs.
- The issue of overfitting due to a small number of training data is addressed by implementing a data augmentation method using data replication.
- Several experiments are conducted to test the DNN’s performance and see how data replication affects data size and localization accuracy.
- Finally, the proposed DNN-based data augmentation strategy (DAS) is compared with traditional Dv-hop algorithms to evaluate its performance.

This article is organized as follows. Section 2 presents the localization process and the calculation of estimated unknown node position. Section 3 describes the data replication based on data augmentation, and the optimal DNN for localization in WSNs is given in Section 4. Simulation and performance analysis are shown in Section 5. Finally, we conclude in Section 6.

### Related Work

Several artificial intelligence (AI)-based approaches across various domains leverage data augmentation techniques to increase dataset size, as extensively discussed in the literature. Data augmentation involves generating slightly modified versions of existing data, with the aim of reducing overfitting in machine learning models by combining both the original and augmented datasets [18].

In fields such as image classification and recognition, data augmentation techniques are widely employed to expand datasets. These methods typically involve replicating the original images and applying transformations such as flipping, cropping, rotation, color adjustments, and the introduction of noise. Each technique serves to diversify the training data and improve model generalization. Flipping, for example, is a simple yet effective method where images are mirrored along a specific axis to create new examples. However, it may not be suitable for datasets containing highly specific or unique characteristics, where such transformations could introduce errors [18]. Cropping, another common technique, involves cutting random portions of the original image and resizing them to a uniform size. This method adds further diversity to the training set by simulating different perspectives or focal points within the data [19].

Rotation augmentation involves rotating an image around an axis by a specific angle, typically between 1 and 20 degrees, depending on the task. The effectiveness of this technique can vary based on the degree of rotation. For example, small rotations are often used in digital recognition tasks, such as in the classification of handwritten digits (MNIST), to increase the training dataset and improve model accuracy [20]. In aircraft classification research, geometric augmentation techniques like cropping, rotating, resizing, and polygon occlusion were applied to original images to introduce variety. The combination of cropping with occlusion led to the most notable performance improvement, enhancing task accuracy by 9% compared to standard methods [21].

Another commonly used augmentation technique is the addition of noise. This method involves introducing random values, often sampled from a Gaussian distribution, into the dataset. For instance, in studies utilizing datasets from the UCI repository (such as heart disease and breast cancer diagnostic datasets), adding Gaussian noise helped convolutional neural networks (CNNs) learn more reliable features, resulting in improved model performance [22]. Different types of noise—such as Gaussian, salt and pepper, and speckle noise—are commonly used to enhance the robustness and performance of deep neural networks across various image-related tasks [23]. One study on Quantitative Structure–Activity Relationships (QSARs) demonstrated the impact of data augmentation using Gaussian noise. The researchers replicated their training data multiple times, each time applying Gaussian noise with different intensities. This approach led to significant improvements in the models’ predictive performance, with accuracy gains of 10–15% across random forest, gradient boosting machine, and support vector machine algorithms [24].

In the context of wireless sensor networks (WSNs), data augmentation has been applied to improve node localization. Some researchers have developed intelligent algorithms to increase training data size by generating virtual anchor nodes around real ones, thereby overcoming the limitations of small datasets and improving the accuracy of deep neural network (DNN) models [25].

Building on these approaches, our work introduces an intelligent data augmentation method specifically designed to address the issue of limited training data for DNNs in WSNs. We replicate the original training data—estimated positions of unknown nodes—multiple times, adding Gaussian noise to each replication. This technique not only increases the dataset size but also introduces diversity, helping the model generalize better and ultimately enhancing its performance in node localization tasks.

## 2. Background

### 2.1. Dv-hop Algorithm

To estimate the positions of unknown nodes, we utilize a multi-hop communication technique through the Dv-hop algorithm [13]. The algorithm operates by calculating the minimum hop count between unknown nodes and anchor nodes. Initially, all anchor nodes broadcast their location information throughout the network. Each anchor node starts with its hop field set to 0, indicating no hops.

As anchor messages propagate across the network, each node records the minimum number of hops needed to reach each anchor node. Upon receiving a message, a node updates its hop count to an anchor only if the new message has a lower hop count than any previously recorded. If a message contains a higher hop count, the node discards it. This process continues iteratively as anchor messages propagate, with each subsequent hop increasing the hop count by one. This ensures that all nodes in the network have the smallest hop counts to each anchor.

**Figure 1:**
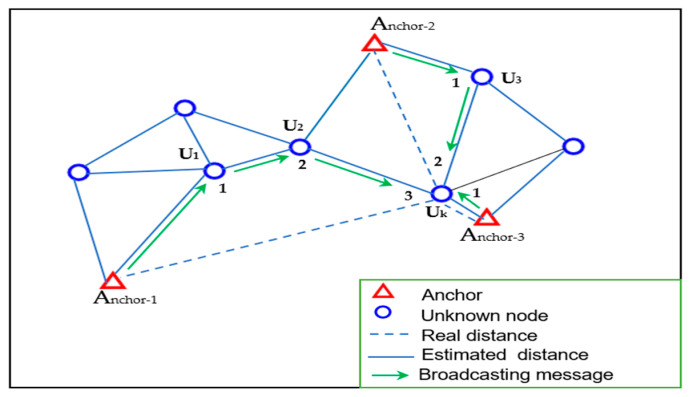
Calculation of the number of hops and corresponding distance.

As shown in Figure 1, where the hop distance between any two neighboring nodes is one, the unknown node U_k_ calculates its minimum hop distance to each anchor node. In this example, U_k_ is three hops from anchor-1, two hops from anchor-2, and one hop from anchor-3. These minimum hop counts serve as the foundation for the following steps in the Dv-hop algorithm, leading to more precise estimation of the unknown node’s position.

This step-by-step broadcasting and hop count updating process ensures that the unknown nodes collect accurate hop information, which is crucial for determining their locations with improved precision in wireless sensor networks.

#### Calculation of Estimated Unknown Node Position

In the second stage, each anchor computes the Euclidean distance $d_{\mathrm{ij}}$ between anchors i and j as follows:

(1) $$d_{\mathrm{ij}}=\sqrt{{\left(x_{i}-x_{j}\right)}^{2}-{\left(y_{i}-y_{j}\right)}^{2}}\;,$$

where $\left(x_{i},y_{i}\right),\left(x_{j},y_{j}\right)$ are known coordinates for anchors i and j, respectively.

The average hop distance that anchor i computes is given by the following formula:

(2) $${\mathrm{AvgHopDis}}_{i}=\frac{\sum_{i\neq j}d_{\mathrm{ij}}}{\sum_{j\neq i}h_{\mathrm{ij}}}$$

where $h_{\mathrm{ij}}$ is the shortest path hop count between anchors i and j.

Then, all unknown nodes have received the average hop distance from anchor nodes which have the least hops between them, and they compute the distance to the anchor nodes based on the two factors of ${\mathrm{AvgDis}}_{i}$ and minimum hop count, denoted as ${\mathrm{hop}}_{i}$. The formula is as follows:

(3) $$d_{i}={\mathrm{hop}}_{i}\times {\mathrm{AvgHopDis}}_{i}$$

The location computation of unknown nodes can be obtained by the following set of equations:

(4) $$\{\begin{array}{c} {\left(\;\hat{x}-x_{1}\right)}^{2}+{\left(\;\hat{y}-y_{1}\right)}^{2}=d_{1}^{2}\\ {\left(\;\hat{x}-x_{2}\right)}^{2}+{\left(\;\hat{y}-y_{2}\right)}^{2}=d_{2}^{2}\\ \vdots \\ {\left(\;\hat{x}-x_{n}\right)}^{2}+{\left(\;\hat{y}-y_{n}\right)}^{2}=d_{n}^{2} \end{array};$$

Subtracting all equations one by one by the last equation in (4), we get the following equation:

(5) $$\begin{array}{c} {\;x}_{1}^{2}-x_{n}^{2}+y_{1}^{2}-y_{n}^{2}-d_{1}^{2}+d_{n}^{2}=2\times \;\hat{x}\times \left(x_{1}-x_{n}\right)+2\times \;\hat{y}\times \left(y_{1}-y_{n}\right)\\ x_{2}^{2}-x_{n}^{2}+y_{2}^{2}-y_{n}^{2}-d_{2}^{2}+d_{n}^{2}=2\times \;\hat{x}\times \left(x_{2}-x_{n}\right)+2\times \;\hat{y}\times \left(y_{2}-y_{n}\right)\\ \vdots \\ {\;x}_{n-1}^{2}-x_{n}^{2}+y_{n-1}^{2}-y_{n}^{2}-d_{n-1}^{2}+d_{n}^{2}=2\times \;\hat{x}\times \left(x_{n-1}-x_{n}\right)+2\times \;\hat{y}\times \left(y_{n-1}-y_{n}\right) \end{array}$$

Each unknown node exploits the stored information to derive its distance $d_{n}$ to the n-th anchor. The coordinates representation of anchor n is $\left(x_{n},y_{n}\right)$, and $\left(\;\hat{x},\;\hat{y}\right)$ are estimated coordinates of the unknown nodes. Equation (5) could be represented by the following equation:

(6) ψC=φ,

where $\psi =2\times \left[\begin{array}{cc} x_{1}-x_{n} & y_{1}-y_{n}\\ \begin{array}{c} x_{2}-x_{n}\\ \vdots \end{array} & \begin{array}{c} y_{2}-y_{n}\\ \vdots \end{array}\\ x_{n-1}-x_{n} & y_{n-1}-y_{n} \end{array}\right],$ and $\varphi =\left[\begin{array}{c} x_{1}^{2}-x_{n}^{2}+y_{1}^{2}-y_{n}^{2}-d_{1}^{2}+d_{n}^{2}\\ x_{2}^{2}-x_{n}^{2}+y_{2}^{2}-y_{n}^{2}-d_{2}^{2}+d_{n}^{2}\\ \begin{array}{c} \vdots \\ x_{n-1}^{2}-x_{n}^{2}+y_{n-1}^{2}-y_{n}^{2}-d_{n-1}^{2}+d_{n}^{2} \end{array} \end{array}\right],$

(7) $$C=\left[\begin{array}{c} \;\hat{x}\\ \;\hat{y} \end{array}\right]={\left(\psi^{T}\psi \right)}^{-1}\psi^{T}\varphi ,$$

where $C=\left[\begin{array}{c} \;\hat{x}\\ \;\hat{y} \end{array}\right]$ is the estimated coordinates of the unknown nodes.

## 3. Localization Process Using Data Replication-Based DNN

**Figure 2:**
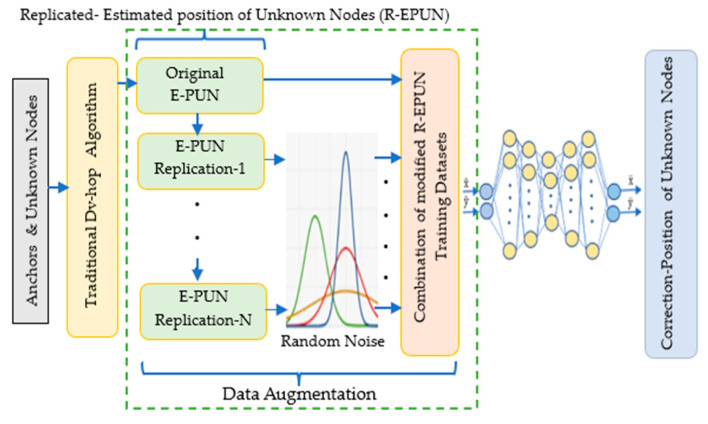
Localization process using data replication-based DNN.

The localization process in wireless sensor networks (WSNs) involves estimating distances and calculating the positions of unknown nodes relative to anchor sensors. In this study, we aim to improve localization accuracy through a multi-phase approach, with the primary contribution indicated by the square dashed lines in Figure 2. The process begins with the random deployment of unknown nodes and anchor sensors within a defined area. Using the Dv-hop algorithm, we initially estimate the positions of these unknown nodes based on the minimum hop counts and computed distances from anchor nodes.

However, one of the main challenges in training deep neural networks (DNNs) for localization tasks is the limited availability of high-quality training data. To address this limitation, we implement a data augmentation strategy that focuses on enhancing the dataset size. This strategy involves replicating the originally estimated positions of the unknown nodes multiple times and introducing Gaussian noise to each replication. By applying Gaussian perturbations, we create slight variations in the dataset, which improves the diversity of the training data.

The augmented dataset, composed of both the original estimated node positions and the modified replications, significantly increases the volume of training data. This expansion is crucial for minimizing overfitting and improving the generalization capacity of the DNN model. In the final step, the augmented dataset is used to train the DNN, which is specifically designed to refine and correct the estimated positions of the unknown nodes, thereby enhancing localization accuracy in WSNs.

### Data Augmentation-Based Data Replication

To enhance the quantity and diversity of training data, we apply a data augmentation strategy through data replication, extending approaches found in various fields [18,19,20,21,22,23,24,25]. This process replicates the initial estimated positions of unknown nodes multiple times. Each replicated dataset is then modified by adding random noise, which introduces variations and thus increases dataset diversity. By combining these modified replicas with the original data, we effectively augment the training dataset.

For example, consider a scenario where k unknown nodes are randomly deployed within a square area. The estimated positions of these unknown nodes (EPUNs) are initially calculated using the Dv-hop algorithm. The Dv-hop process is described in Equations (5)–(7), where each node first receives the minimum hop count values for each anchor node. The anchor nodes broadcast their positions, which are propagated throughout the network. Each node records the smallest number of hops to each anchor and discards any subsequent messages from the same anchor if they carry a higher hop count. With every hop increment during message propagation, all nodes eventually record the minimal hop counts to every anchor node.

Once the estimated positions are obtained, the data augmentation process begins. We replicate these estimated positions several times, applying random Gaussian noise to each replication to create a more diverse training dataset. This expanded and augmented dataset is then used to train the deep neural network (DNN). The DNN leverages this augmented data to refine and adjust the estimated positions of the unknown nodes, leading to improved localization performance in WSNs more accurately.

(8) $$\mathrm{EPUN}={\left[\begin{array}{c} {\hat{x}}_{1}\;{\hat{x}}_{2}\;\ldots \ldots \ldots {\hat{x}}_{k}\;\\ {\hat{y}}_{1}\;{\hat{y}}_{2}\ldots \ldots \ldots {\hat{y}}_{k} \end{array}\right]}_{2\times k}$$

**Figure 3:**
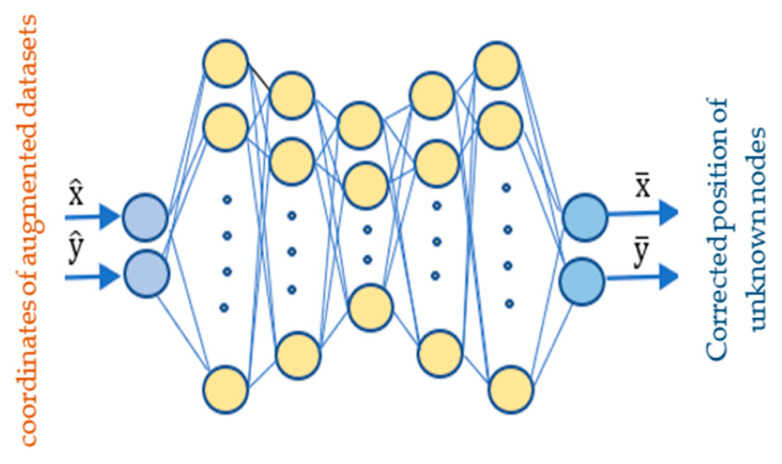
Proposed DNN architectural model.

In this scenario, we describe the implementation of data augmentation where the estimated positions of unknown nodes (EPUNs) are replicated multiple times, as illustrated in Figure 3. This process involves creating multiple copies of the original EPUN dataset and then introducing perturbations to these replications. The perturbations are modeled as zero-mean Gaussian random variables with a variance of $\sigma^{2}$, as detailed in Algorithm 1. Specifically, the original EPUN dataset is first replicated several times to increase the quantity of training data. Each of these replicated datasets is then modified by adding Gaussian noise, characterized by a mean of zero and a variance of $\sigma^{2}$. This noise perturbation ensures that the augmented datasets are diverse, thereby enhancing the robustness of the training data.
**Algorithm 1.** Generator for Modification of Replicated Estimated Location of Unknown NodesInput: Node Amount, Unknown Node(UN Amount), Anchor Amount, variance. C (generation of Coordinate of all nodes). Anchor = [C(1,1:AnchorAmount); C(2,1:AnchorAmount)].
dhop      Average hop distance. Hop       Minimum hop count. ED         Estimated distance. MDR     Modified data replication. EPUN   Estimated position of unknown node. X           Coordinate of Unknown Node. $\mathrm{perturbation}\sim N\left(\mu \;=0\;,\frac{\sigma_{\mathrm{perturbation}}^{2}}{2}\;\right)$$.\;\%\;\mathrm{Mean}\;\mu =0\;\mathrm{STD}\;\sim \sigma_{\mathrm{perturbation}}$. Output:
1. % econd stage of Dv-hop algorithm.2. for i = 1:UNAmount3. for j = 1:Anchor Amount4. ED(j,i) = dhop(j,1) × hop(j,i); % Estimated distance Equation (3)5. end6. end7. for i = 1:28. for j = 1:(AnchorAmount-1)9. a(i,j) = Anchor(i,j)-Anchor(i, AnchorAmount);10.end11.end12.A = −2 × (aT);13.for i = 1:UNAmount14.for j = 1:(AnchorAmount-1)15.B(j,1) = ED(j,i)2-ED(AnchorAmount,i)2-Anchor(1,j)2+Anchor(1,AnchorAmount)2-Anchor (2,j)2+Anchor (2, AnchorAmount)2;16.end17.X1 = (AT × A) − 1 × AT × B; % ELUN Equation (7).18.X(1,i) = X1(1,1); % x coordinate19.X(2,i) = X1(2,1); % y coordinate20.end21.% Implementation of data augmentation model.22.for sigma = 0:1:m % m is the total number of repetitions.23.$\mathrm{Perturbation}=\left(\frac{\mathrm{sigma}}{m\times \sqrt{2}}\right)\;$randn(2, UN amount);24.MDR = X + perturbation; % Modification of Estimated coordinates.25.EPUN{ sigma + 1} = MDR.26.TD = cat (2, EPUN {:}); % Training Dataset27.end

Algorithm 1 outlines the specific steps involved in this data augmentation process. It starts with replicating the original EPUN data multiple times. For each replication, Gaussian noise is added to introduce variability. The resulting augmented datasets are then combined with the original data to form a comprehensive and diverse training dataset. This augmented dataset is used to train the deep neural network, thereby improving its ability to accurately predict the positions of unknown nodes in WSNs.

Figure 3 visually represents the data augmentation process, showing the multiple replications of the EPUN and the subsequent addition of Gaussian noise to each replication. This scenario demonstrates how data augmentation can significantly enhance the quality and diversity of the training data, ultimately leading to better performance of the neural network in localization tasks, i.e.,

$$\mathrm{Perturbation}\left[\begin{array}{c} w_{x}\\ w_{y} \end{array}\right]\sim \left[\begin{array}{c} N\left(0\;,\frac{\sigma_{x}^{2}}{2}\right)\\ N\left(0\;,\frac{\sigma_{y}^{2}}{2}\;\right) \end{array}\right]$$

The modified EPUN for k unknown nodes represented as follows:

(9) $$\mathrm{Modified}\_\mathrm{EPUN}=\left[\begin{array}{c} {\hat{x}}_{1}\;{\hat{x}}_{2}\;\ldots \ldots \ldots {\hat{x}}_{k}\;\\ {\hat{y}}_{1}\;{\hat{y}}_{2}\ldots \ldots \ldots {\hat{y}}_{k} \end{array}\right]+\left[\begin{array}{c} w_{1x}{\;w}_{2x}\;\ldots \ldots \ldots {\;w}_{\mathrm{nx}}\;\\ w_{1y}{\;w}_{2y}\ldots \ldots \ldots {\;w}_{\mathrm{ny}} \end{array}\right]$$

(10) $$\begin{array}{c} \mathrm{Original}\mathrm{data}=[\begin{array}{c} {\hat{x}}_{1}\;{\hat{x}}_{2}\;\ldots \ldots \ldots {\hat{x}}_{k}\;\\ {\hat{y}}_{1}\;{\hat{y}}_{2}\ldots \ldots \ldots {\hat{y}}_{k} \end{array}]\;={\mathrm{MDR}}_{0}\\ {\mathrm{Replication}}_{1}=[\begin{array}{c} {\hat{x}}_{1}\;{\hat{x}}_{2}\;\ldots \ldots \ldots {\hat{x}}_{k}\;\\ {\hat{y}}_{1}\;{\hat{y}}_{2}\ldots \ldots \ldots {\hat{y}}_{k} \end{array}]+[\begin{array}{c} w_{1x}{\;w}_{2x}\;\ldots \ldots \ldots {\;w}_{\mathrm{nx}}\;\\ w_{1y}{\;w}_{2y}\ldots \ldots \ldots {\;w}_{\mathrm{ny}} \end{array}{]}_{\mathrm{at}\sigma =1}={\mathrm{MDR}}_{1}\\ {\mathrm{Replication}}_{2}=[\begin{array}{c} {\hat{x}}_{1}\;{\hat{x}}_{2}\;\ldots \ldots \ldots {\hat{x}}_{k}\;\\ {\hat{y}}_{1}\;{\hat{y}}_{2}\ldots \ldots \ldots {\hat{y}}_{k} \end{array}]+[\begin{array}{c} w_{1x}{\;w}_{2x}\;\ldots \ldots \ldots {\;w}_{\mathrm{nx}}\;\\ w_{1y}{\;w}_{2y}\ldots \ldots \ldots {\;w}_{\mathrm{ny}} \end{array}{]}_{\mathrm{at}\sigma =1/2}={\mathrm{MDR}}_{2}\\ \vdots \\ {\mathrm{Replication}}_{m}=[\begin{array}{c} {\hat{x}}_{1}\;{\hat{x}}_{2}\;\ldots \ldots \ldots {\hat{x}}_{k}\;\\ {\hat{y}}_{1}\;{\hat{y}}_{2}\ldots \ldots \ldots {\hat{y}}_{k} \end{array}]+[\begin{array}{c} w_{1x}{\;w}_{2x}\;\ldots \ldots \ldots {\;w}_{\mathrm{nx}}\;\\ w_{1y}{\;w}_{2y}\ldots \ldots \ldots {\;w}_{\mathrm{ny}} \end{array}{]}_{\mathrm{at}\sigma =1/m}={\mathrm{MDR}}_{m} \end{array}$$

(11) $$\mathrm{Training}\;\mathrm{datasets}\;\left({\mathrm{TD}}_{m}\;\right)={\left[{\mathrm{MDR}}_{0};{\;\mathrm{MRD}}_{1};{\;\mathrm{MRD}}_{2}\;;\;\ldots \ldots \;{\mathrm{MRD}}_{m}\right]}_{\left(2\times \left(m+1\right)\right)\times k}$$

The unknown nodes U_k_ are represented as (2×k), where (k = 1, ………, Nu), Nu being the total number of unknown nodes. The σ is the standard deviation (STD) of random noise, with its maximum value set to one. The parameter (m) denotes the total number of data repetitions. While the perturbation is (2×k), its dimension is the same as the original estimated position of the unknown nodes’ dimension.

MDR_0_ represents the original EPUN.

MDR_1_ represents the modified data of the first replication.

MDR_2_ represents the modified data of the second replication.

MDR_m_ represents the modified data of the last replication.

The total size of the training data, TD_m_, is determined by the combination of the original estimated positions and the modified replicated datasets. This comprehensive dataset enhances the quantity and diversity of the training data, which is crucial for improving the accuracy of the deep neural network in localizing unknown nodes.

For testing, the effect of the combination datasets for training the DNN is outlined in Table 1.

## 4. Proposed DNN for Localization in WSNs

In the field of node localization for wireless sensor networks (WSNs), various machine learning (ML) techniques have been explored. This article specifically focuses on Deep Neural Networks (DNNs) due to their exceptional ability to capture complex input/output relationships, making them highly effective for localization tasks. Building on the methodology described in [25], we have implemented an optimized DNN architecture-based localization algorithm.

DNNs are particularly advantageous because they can combine the outputs of independently trained neural networks, thereby enhancing the overall model performance. To minimize localization error, we conducted a comprehensive exploration of different DNN architectures. This involved experimenting with various combinations of hidden layers and neurons through a multi-iterative process.

To achieve a low localization error, the chosen number of hidden layers and neurons was accomplished by training multiple different DNN architectures. Hence, the number of hidden layers and neurons was increased to obtain the best DNN performance, since the node localization requires a lower error and higher correlation coefficient between estimated and actual locations. First, the hidden layer was executed by changing the number of neurons from (5-5-2-5-5, 5-5-5-5-5, 10-5-5-5-10, and 5-10-5-10-5), as shown in Figure 3. Based on the DNN performance, we noticed that the mean square error (MSE) value of the DNN training was unsatisfactory. Therefore, the number of neurons in hidden layers was changed to constitute the minimum MSE relative to the other DNN architectures. In this work, the finalized architecture consists of the following: an input layer that receives the coordinates of the augmented datasets; five hidden layers with neuron counts of 20, 10, 5, 10, and 20; an output layer that provides the corrected positions of the unknown nodes. Figure 3 shows the architecture of the DNN that was adopted. The DNN was selected to improve the localization accuracy. For training, testing, and validation purposes, we utilize the coordinates of the augmented datasets. This dataset is divided into three subsets, 70% for training, 15% for testing, and 15% for validation, following established practices [25].

Levenberg–Marquardt backpropagation was used for training our model, where hyperbolic tangent sigmoid activation function has been used for the hidden layers, while a pure line function has been used in the output layer to provide the best output [26].

## 5. Performance Analysis

In this section, we analyze how well the proposed DNN localization algorithms perform. We start by describing a two-dimensional simulated system model and explain how our model can improve localization accuracy. Finally, we present the performance results and discuss them.

### 5.1. Simulated System Model

Our simulated network consisted of nodes deployed within a 100 × 100 m^2^ square area, with each node having a uniform transmission range. For all simulations, sensor nodes (ranging from 50 to 300 nodes) were randomly deployed, with configurations of 50, 100, 150, 200, 250, and 300 nodes. All other system parameters were kept constant at their default values, as outlined in Table 2.

**Figure 4:**
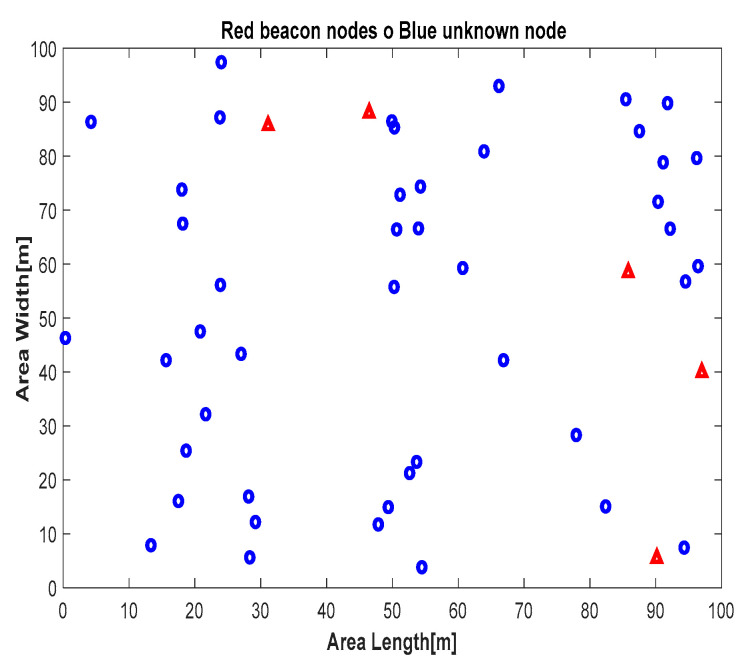
Distribution of 50 nodes. 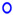: unknown nodes, 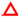: anchors.

**Figure 5:**
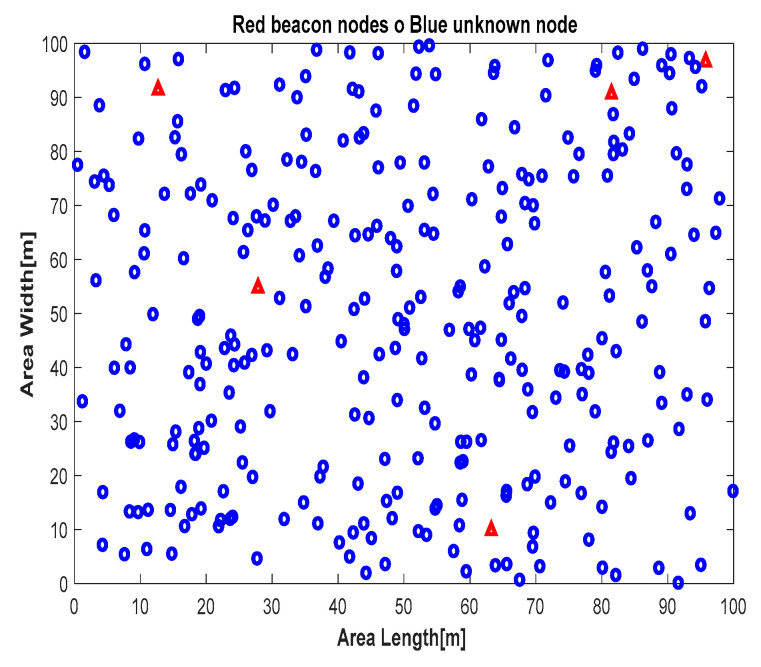
Distribution of 300 nodes. 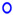: unknown nodes, 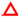: anchors.

As an example, the first scenario involved 50 nodes (5 anchor nodes and 45 unknown nodes) randomly placed in the area, as shown in Figure 4. The final scenario, involving 300 nodes (5 anchor nodes and 295 unknown nodes), is illustrated in Figure 5. For each configuration, the proposed localization algorithms were simulated using the same network layout to enable a consistent performance comparison. The results reported in this paper are based on a thousand simulation runs for each tested configuration.

### 5.2. Simulation Results

To evaluate the performance of the proposed algorithm, a series of simulations was conducted using DNNs across different node configurations: specifically, scenarios involving 50, 100,150, 200, 250, and 300 nodes. These simulations were executed with varying sizes of training datasets, meticulously detailed in Table 3.

The primary evaluation metric used in these comparisons was the normalized root mean square error (NRMSE), expressed in Equation (12). This metric serves as a critical indicator of localization precision, quantifying the average discrepancy between predicted and actual node positions normalized by the range of node positions.

By systematically adjusting both the number of nodes and the scale of training datasets, our simulations aimed to uncover how these variables impact the algorithm’s performance. This detailed analysis provided valuable insights into the algorithm’s robustness and its ability to achieve accurate localization across different WSN setups and dataset sizes.

(12) $$\mathrm{NRMSE}=\frac{\sum_{i=0}^{U}\sqrt{{\left(x_{i}-{\;\hat{x}}_{j}\right)}^{2}+{\left(y_{i}-{\;\hat{y}}_{j}\right)}^{2}}}{\left(N_{U}\times R\right)}$$

where $\left(x_{i},y_{i}\right)$, and $\left({\;\hat{x}}_{j},{\;\hat{y}}_{j}\right)$ represents the real position, and estimated position of the unknown node, while the parameters Nu and R are defined in Table 2.

#### 5.2.1. Effect of Data Replication and Number of Nodes on Localization Error

**Figure 6:**
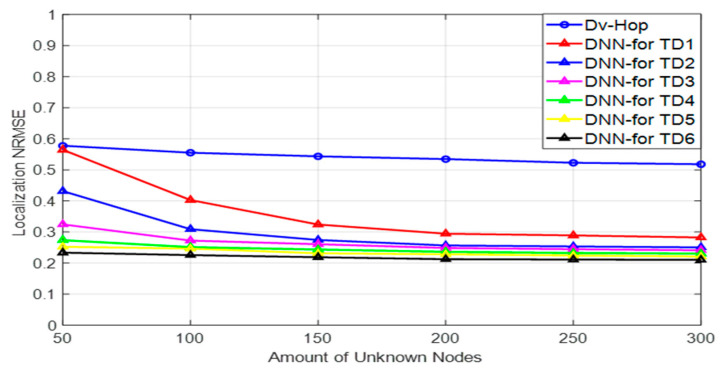
NRMSE performance vs. unknown number of nodes.

The comparison of the localization accuracy, measured using NRMSE, for two algorithms, Dv-hop and the proposed DNN model based on the data augmentation algorithm, is shown in Figure 6. The performance of the DNN model was evaluated using different sizes of training data, labeled as TD1 to TD6, as described in Table 3.

- Effect of Dv-hop algorithms.

As shown in Figure 6, the DV-Hop algorithm achieved relatively low localization accuracy across different unknown node configurations, primarily because its performance is significantly influenced by the number of anchor nodes and the communication range. While increasing the number of anchors generally improves localization accuracy, it also leads to higher energy consumption, which is a critical concern in wireless sensor networks. Additionally, deploying many anchors can be cost-prohibitive and impractical in many real-world applications.

Another challenge with the Dv-hop algorithm is the availability of sufficient training data, especially when using a limited number of anchors. To address this issue, we introduced a data augmentation strategy (DAS) that replicates the original datasets, thereby reducing the need for many anchors. By augmenting the training data, we mitigate the prohibitive costs associated with deploying numerous anchors while maintaining or even improving localization accuracy. This approach ensures that the DNN model can perform effectively even with a limited number of anchors.

**Figure 7:**
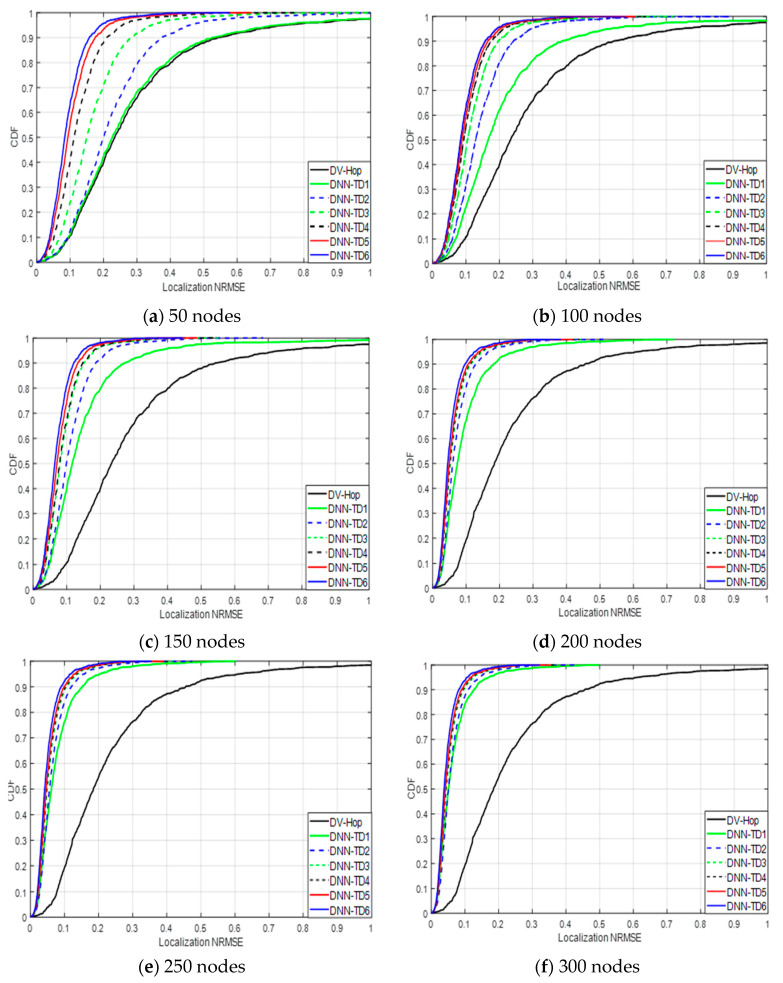
CDFs of localization NRMSE for Dv-hop algorithm and DNN at different value data sizes.

In the current comparison, as detailed in Table 4 and Figure 7, the Dv-hop algorithm was evaluated across different network configurations with varying numbers of sensor nodes but with a fixed number of anchor nodes (five anchors). The results reveal that the Dv-hop algorithm achieved relatively low localization accuracy when fewer anchors were used, especially in larger networks.

The core limitation of the Dv-hop algorithm is the trade-off between accuracy and the number of anchor nodes. With only five anchors, the algorithm’s accuracy diminishes, as fewer reference points lead to less reliable distance estimates based on hop counts. This issue becomes more pronounced in larger networks, where the performance of the Dv-hop algorithm deteriorates further, resulting in higher NRMSE values.

Although increasing the number of anchors could potentially improve accuracy, it introduces additional challenges. Deploying more anchors involves higher costs and increased energy consumption, which can be prohibitive for large-scale implementations. Consequently, while the Dv-hop algorithm might perform better with more anchors, its practical application is limited by the associated costs and energy requirements.

This comparison underscores the limitations of the Dv-hop algorithm and highlights the advantages of alternative approaches (proposed DNN model with data augmentation), which can achieve better localization accuracy without the drawbacks of higher costs and energy consumption.

- Training Data Impact (TD1 to TD6).

The DNN model’s localization accuracy was analyzed with varying amounts of training data (from TD1, the smallest set, to TD6, the largest set). These different data sizes allowed for a thorough evaluation of the model’s ability to generalize across different network sizes and configurations.

- Comparison Across Network Configurations.

Both the Dv-hop algorithm and the proposed DNN model were evaluated across various network configurations to compare their performance in terms of localization accuracy. This comparison was conducted using different numbers of sensor nodes, with accuracy measured by the NRMSE.

Setup 1: 50 Nodes (45 Unknown Nodes, 5 Anchors)

In this initial setup, 45 unknown nodes and 5 anchor nodes were used, as detailed in Table 4. With the smallest training dataset (TD1), the DNN’s accuracy was comparable to that of the Dv-hop algorithm. This similarity is likely due to the limited amount of training data available in TD1. However, as additional data were progressively introduced (TD2 to TD6), the DNN’s accuracy improved significantly, eventually surpassing the Dv-hop algorithm. This improvement demonstrates that the DNN model benefits from larger datasets, enhancing its performance and exceeding the traditional Dv-hop method when provided with sufficient training data.

Setup 2: 100 to 150 Nodes

For network configurations involving 100 to 150 nodes, Table 4 shows that the DNN model initially performed slightly better than the Dv-hop algorithm with smaller training datasets. Despite this, the limited data in TD1 and TD2 led to some overfitting. As the size of the training dataset increased from TD2 to TD6, the DNN model’s accuracy improved substantially. This improvement indicates that the DNN model’s performance benefits from additional data, allowing it to generalize better and achieve higher accuracy in larger networks compared to the Dv-hop algorithm.

Setup 3: 200, 250, and 300 Nodes

In setups with 195, 245, and 295 unknown nodes (and 5 anchor nodes), the DNN model demonstrated strong performance even with the original, smaller training data (TD1), as illustrated in Figure 6. The larger number of nodes provided sufficient data diversity, which helped prevent overfitting early on. As the training data size increased from TD2 to TD6, the DNN’s accuracy continued to improve, showcasing the model’s scalability and effectiveness in handling larger networks.

Overall, the experiments revealed that the proposed DNN model based on data augmentation consistently outperformed the traditional Dv-hop algorithm. The DNN model showed significant improvements in accuracy as both the number of nodes and the size of the training dataset increased, highlighting its superior performance and adaptability in varying network conditions.

#### 5.2.2. Cumulative Distribution Function vs. Localization NRMSE

It is also useful to analyze the system performance by using the cumulative distribution function (CDF) of a performance metric for having a better insight into the system behavior. We have used it for some of our simulation results for better understanding the performance of the proposed localizing algorithms. The CDFs of the localization NRMSE with different training sizes at different numbers of nodes (50, 100, 150, 200, 250, and 300 nodes) are illustrated in Figure 7.

Figure 7a presents the cumulative distribution function (CDF) of the localization NRMSE for a setup with 45 unknown nodes and 5 anchor nodes (totaling 50 nodes). Using the Dv-hop algorithm, only 40% of sensors were able to estimate their positions with an NRMSE below 0.2. However, when applying our proposed DNN model trained with datasets of increasing sizes (from TD1 to TD6), the percentages of sensors estimating positions with an NRMSE below 0.2 improved significantly to 40%, 53%, 76%, 90%, 92%, and 95%, respectively.

From Figure 7b to Figure 7f show the CDF of localization NRMSE for setups with 100, 150, 200, 250, and 300 nodes. With the Dv-hop algorithm, only 40% to 53% of sensors were able to estimate their positions with an NRMSE below 0.2. In contrast, when using the proposed DNN trained with datasets of varying sizes (from TD1 to TD6), the percentages increased significantly, ranging from 62% to 98%. This indicates that a higher percentage of sensors could estimate their positions with an NRMSE below 0.2 when using the DNN compared to the Dv-hop algorithm.

At lower node counts, the results show that with the original training data, the DNN performed similarly to the Dv-hop method in estimating unknown node positions, likely due to the limited amount of training data. However, as more training data were added (from TD1 to TD6), the accuracy of the DNN in estimating node locations improved significantly. Additionally, as the number of nodes increased, the DNN’s accuracy improved markedly, outperforming the Dv-hop algorithm. These findings demonstrate the effectiveness of the proposed algorithm, particularly in scenarios with a low number of nodes.

## 6. Conclusions

This article presents a data augmentation approach based on a data replication method to enhance the performance of deep neural networks (DNNs) for range-free localization using the Dv-hop algorithm in wireless sensor networks (WSNs) in Internet of Things (IoT) applications. WSN deployments with a limited number of nodes often result in constrained datasets for training DNNs, leading to reduced localization accuracy. To address this limitation, we proposed a technique to augment the training data by generating multiple replicas of the original dataset and introducing Gaussian noise to each replica to create modified versions. The combination of the original and modified datasets significantly expands the training set, improving the DNN’s ability to accurately localize nodes.

Experiments were conducted using DNNs across different node configurations, and the performance of the DNN was evaluated using normalized root mean square error (NRMSE) metrics. Initially, the localization accuracy of the DNN improved as the number of nodes in the network configurations increased. Additionally, the DNN’s performance with varying training data sizes was compared across different node configurations.

Further analysis using the cumulative distribution function (CDF) of the NRMSE highlighted the significant impact of the proposed data augmentation strategy, as the DNN accurately estimated the positions of the majority of sensor nodes when trained on a combination of multiple datasets. However, the experiment shows that the data augmentation technique significantly improved the performance of the DNN, especially in scenarios with a lower number of nodes. The proposed DNN-based data augmentation strategy effectively addresses the challenges associated with limited training data and offers significant improvements over traditional Dv-hop algorithms as the training dataset grows. This approach provides a promising solution for enhancing localization accuracy in WSNs and represents a significant advancement in the field of IoT.

## Figures and Tables

**Table 1:** Combination of modified data replication (MDR).

No. of Repetitions	Training Data (TD)	Data Combination
0	TD_1_	[MDR_0_]_2×k_
1	TD_2_	[MDR_0_, MDR_1_]_4×k_
2	TD_3_	[MDR_0_, MDR_1_, MDR_2_]_6×k_
m	TD_m_	[MDR_0_, MDR_1_, MDR_2_,……, MDR_m_]_(2×(m+1))×k_

**Table 2:** Simulated system parameters used in different experiments.

	Contents of Experiments	
N	Number of nodes	50; 100; 150; 200, 250; 300
Nu	Number of unknown nodes	45; 95; 145; 195; 245; 295
A	Number of real anchors	5
Sa	Square area	100 × 100 m^2^
R	Ccommunications range	30 m
ρ	Node density	0.01

**Table 3:** Training data size for nodes (unknown nodes +5 anchors).

Training Data	50	100	150	200	250	300
TD1	2 × 45	2 × 95	2 × 145	2 × 195	2 × 245	2 × 295
TD2	4 × 45	4 × 95	4 × 145	4 × 195	4 × 245	4 ×295
TD3	6 × 45	6 × 95	6 × 145	6 × 195	6 × 245	6 × 295
TD4	8 × 45	8 × 95	8 × 145	8 × 195	8 × 245	8 × 295
TD5	10 × 45	10 × 95	10 × 145	10 × 195	10 × 245	10 × 295
TD6	12 × 45	12 × 95	12 × 145	12 × 195	12 × 245	12 × 295

**Table 4:** Result of number of nodes vs. Dv-hop and DNN at different data sizes.

	Nodes	50	100	150	200	250	300
	Dv-hop	57.77%	55.52%	54.35%	53.46%	52.28%	51.81%
	DNN-for TD1	52.23%	40.28%	32.40%	29.45%	28.89%	28.23%
	DNN-for TD2	35.15%	30.90%	27.43%	25.66%	25.34%	25.04%
NRMSE	DNN-for TD3	32.44%	27.23%	26.06%	24.85%	24.46%	24.11%
	DNN-for TD4	27.36%	25.12%	24.34%	23.62%	23.23%	23.03%
	DNN-for TD5	25.23%	24.66%	23.14%	22.83%	22.43%	22.12%
	DNN-for TD6	23.34%	22.56%	21.87%	21.23%	21.12%	21.03%

## Data Availability

Data sharing is not applicable to this article.
